# An understanding of the motivations that influence the beef cattle production systems adopted by farmers in central Mozambique

**DOI:** 10.1371/journal.pone.0325929

**Published:** 2025-06-17

**Authors:** Télis Adolfo Cumbe, Benedito Armando Cunguara, Concepta Margaret McManus, Antónia Mendes Paizano Alforma, Júlio Otávio Jardim Barcellos

**Affiliations:** 1 Department of Animal Science, Federal University of Rio Grande do Sul (UFRGS), Porto Alegre, Rio Grande do Sul, Brazil; 2 Faculty of Agricultural Science, Zambezi University (UniZambeze), Ulónguè, Tete, Mozambique; 3 Ministry of Economy and Finance, Development Cabinet of Compact II, Mozambique; 4 Faculty of Agronomy and Veterinary Medicine, University of Brasília, Brasilia, Brazil; 5 Estação Zootécnica de Angónia (EZA), Centro Regional da Zona Centro, Instituto de Investigação Agrária de Moçambique (IIAM), Ulónguè, Tete, Moçambique; Universidade Federal de Mato Grosso do Sul, BRAZIL

## Abstract

The analysis of factors that affect livestock production, from the farmers’ perspective, is essential for improving efficiency in animal production. The objectives of this study were to analyse: i) the historical and current motivations for beef cattle production and ii) the situation of communal pasture areas. For data collection, semi-structured interviews were held with one hundred and one farmers in the districts of Angónia, Changara and Manica, in Mozambique. The results show that in Angónia and Changara districts, the primary motivation for starting beef cattle production was to keep cattle as a saving asset, whereas the primary motivation of Manica’s farmers was to use cattle as draught animal power to expand crop production. These motivations remain the same over the years of the farmers’ experience. Grazing areas have decreased over the years, mainly due to their occupation for crop production, and this perception was associated with the studied district (*p* = 0.004). The studied districts, particularly Angónia and Manica, have similar characteristics, suggesting that similar intervention models may be designed. The results raise the question of how to increase the productivity of beef cattle systems, primarily motivated by savings and animal traction while maintaining their characteristics, which are essential for the socio-economic conditions of the farmers. Overall, the study suggests that motivations for beef cattle production and the establishment of grazing areas should be considered when developing strategies and policies to improve these systems.

## 1 Introduction

Increasing livestock productivity continues to be one of the major challenges to meet the demand for meat [1]. Livestock production is essential for supplying meat and milk for human nutrition, accounting for 34% of the animal protein and 18% of the dietary energy consumed globally [2]. It also plays a significant role in the livelihood of several populations [3].

The contribution of cattle production to Mozambique’s economy is considered incipient [4–5] as cattle production is based on a herd of 2,183,857 heads, which are reared in underdeveloped systems and on small farms (≤ 10 head of cattle/household [6,7]. Despite the low total cattle meat production, estimated at 14,886 metric tons (0.53 kg per capita) [8], the beef production system and its animal protein supply function are vital for the country. The number of cattle heads increased since 1961, with a 58.2% relative growth rate between 2010 and 2018 and 12.4% between 2014 and 2018 [9,10]. However, the technological level of Mozambique’s animal production systems is low, leading governmental and non-governmental organisations to prioritise policies and actions to improve such systems.

The strategic plans for agricultural development in Mozambique (2010–2019) [5] try to address this concern, but the proposed actions have not achieved the required development of the country’s cattle production systems [11]. Those results may be primarily attributed to the lack of adaptation of policies to the existing production systems. Policies and strategies are usually based on studies that do not include information that considers the farmer’s attitude towards adopting techniques that may improve their production system [12].

In this study, based on motivational approaches [13–15], we use the terminology “motivations” of cattle farmers to refer to why farmers raise cattle over the years of their experience. In this context, the benefits of animals such as savings, animal traction, source of manure, and even social prestige in some communities [11], are included as motivations. The analysis of these motivations, with emphasis on the historical motivation to start rearing cattle and the characteristics of the applied production systems, is important because it will allow understanding the gap between the country’s objectives of improving cattle production and the farmers. Therefore, their accurate description is essential when recommending technical modifications, extension service initiatives, and policy changes [16,17].

The objectives of this study were to analyse: i) the historical and current motivations for beef cattle production and ii) the situation of communal pasture areas.

## 2 Materials and methods

### 2.1 Study area

The study was carried out in two different districts of Tete province (Angónia and Changara districts) and one of Manica province (Manica district), located in central Mozambique. The main characteristics of beef cattle systems selected for evaluation are shown in Table 1. In all districts, agriculture is the primary economic activity developed by the local population. The districts were selected based on three principles: (1) the large herd size of central Mozambique [7]; (2) the presence of the three main cattle breeds of Mozambique (*Angone, Bovino de Tete*, and *Landim,* which are found in the districts of Angónia, Changara, and Manica, respectively) [18] and (3) their different agro-climatic conditions, which determine the capacity for farming activities.

**Table 1:** Key characteristics ^A^ and number of respondents interviewed in the studied districts of central Mozambique.

Location	Area, km^2^	Annual rainfall (mm)	Average annual temperature (Min.-Max.), °C	^b^ Beef cattle, n	^c^ Cattle per capita	Number of respondents
Angónia	3272	1100 - 1200	18 - 22	33864	0.08	33
Changara	8660	644	20.5 - 32.5	53782	0.24	37
Manica	4594	1000 - 1020	14 - 28.4	35978	0.14	31

^A^ Angónia [19], Changara [20], and Manica [21].

^b^ Total head of cattle [7].

^c^ Determined based on the 2020 population estimate in each district.

### 2.2 Data collection on beef cattle farmers

Interviews were held between 26 September 2021 and 14 January 2022, using a semi-structured questionnaire with beef cattle farmers from the districts of Angónia, Changara and Manica. To this and, using a multistage sampling method, the farmers were randomly selected and identified with the aid of livestock sector representatives from studied districts. In the multistage sampling procedure, the main selection criteria were that the farms should not be near each other and represent the production issues faced in each district. Previous field observations have shown that farmers living in the same community or near each other tend to respond similarly due to their mutual influence on cattle production habits or understanding of this activity. In the selection, farmers with a minimum experience of five years were included. Interviewees were informed of the study’s purpose, and their verbal consent was requested before the face-to-face interviews were applied. The farmers’ consent was witnessed by livestock representatives from the studied districts. This process was preceded by the official approval of the study by the livestock authorities in the districts of Angonia, Changara, and Manica. A total of one hundred and one questionnaires were validated (Angónia, n = 33; Changara, n = 37; Manica, n = 31). The minimum sample size of 99 farmers was calculated using the simplified Yamane formula, which assumes a significance level of 95% and a proportion of 0.5 (p) of the study population [22] (Eq. 1)


$$\begin{array}{c} n=N/\left[1+N(e{)}^{2}\right] \end{array}$$ (1)

Where: n – is the sample size, e – is the margin of error, and N – is the population size.

A pre-test was applied to sixteen farmers. The pre-test was conducted in all districts due to their dialectal differences. The pre-test also allowed us to readapt the questionnaire, particularly the question related to ranking the primary motivations for beef cattle production. The methodology proposed by FAO was applied [23], generating pictures with text depicting animal use for draught animal power, milk, savings, manure, and meat. The images allowed an easier understanding of the interviewees when requested to rank their motivations in order of importance.

### 2.3 Data analysis

The districts were considered independent variables in all analyses except beef cattle production motivation. One-way analysis of variance was applied to quantitative data, which included the number of pigs, goats, chickens, and other species on the farm. Variables that did not present normal distribution and equal variances, such as household size, experience in cattle production (number of years in cattle production), and cattle head number at the start of production, were analysed by the Kruskal-Wallis test [24].

The scores assigned by the farmers for their current motives for producing beef cattle and the number of family members and workers involved in grazing activities were analysed by the Kruskal-Wallis test. When significant (*p* < 0.05), variables were compared by the Mann-Whitney U *post hoc* test.

The chi-square test (χ^2^) was applied to test possible associations of qualitative data and those classified in ranges (farmers’ education level and age, and herd size) with the different districts, and when significant (*p* < 0.05), the results were compared to identify the proportional differences after the Bonferroni correction [24].

The variables funding sources to pay beef production costs, motivation for starting beef cattle production, and causes of grazing area decrease were converted into binary numbers to allow χ^2^ application, aiming to determine the effect of each of the main characteristics described in the three variables on the studied districts. The main characteristics were (*i*) sale of crop products (= 1) versus other sources (= 0) as funding sources to pay cattle production costs, (*ii*) savings (= 1) versus other motives (= 0) as motivation for starting beef cattle production, and (*iii*) occupation for crop production (= 1) versus other causes (= 0) of decreasing grazing areas.

Additionally, the variables farmers’ age, experience in cattle production, initial and current herd size, and education level were submitted to the Mann-Whitney U test to understand how differences in farmer motivations influence the beef cattle production system. All data were analysed using the software SPSS, version 21.

## 3 Results

### 3.1 Socio-demographic aspects and experience of the beef cattle farmers

The main characteristics of the farmers are shown in Table 2. Out of the total farmers interviewed, over 94% were male. Farmers’ age range differed among districts (*p* = 0.039), with a higher proportion of young farmers in Angónia (<41 years) compared with Manica. Household sizes differed among districts, with Angónia having the lowest median. In all districts, crop production is the most important farming activity, followed by livestock production.

**Table 2:** General socio-demographic characteristics of beef cattle farmers in the studied districts of central Mozambique.

	Angónia (n = 33)	Changara (n = 37)	Manica (n = 31)	χ^2^	*p*-value
Gender of household head, %					
Male	96.97	94.6	100		
Female	3.03	5.4	0.0		
Age of household head (year), %					
<41	33.33^a^	21.62^ab^	6.45^b^	13.28	0.039
41–50	27.23^a^	32.43^a^	32.26^a^		
51–60	30.30^a^	13.51^a^	32.26^a^		
>61	9.09^a^	32.43^a^	29.03^a^		
Household size (people)^D^					
Mean (SD)	5.89 (2.05)	9.28 (3.75)	8.65 (3.24)		
Median (Q1-Q3)	6^a^ (5–7)	9^bc^ (7–9)	9^c^ (7–12)		<0.001
Education level (%)					
Illiterate	6.06	2.70	0.00	10.32	0.289^***^
Low primary (grades 1–5)	30.30	45.95	38.71		
Upper primary (grades 6–7)	36.36	37.84	29.03		
Junior secondary (grades 8–10)	21.21	8.11	32.26		
Pre-university (grades 11–12)	3.03	2.7	0.00		
University degree	3.03	2.7	0.00		
Main sources of household income (%)					
Crops	78.79	70.27	80.65	3.41	0.814^***^
Livestock	6.06	5.41	3.23		
Crops and Livestock equally	0.0	8.11	3.23		
Other activities	15.15	16.22	12.9		

SD: standard deviation; Q1-Q3: quartiles 1 and 3.

^D^ Father, mother, children, and grandparents.

*** Results of the Fisher’s exact test.

^abc^ Values without a common superscript letter in the same row differ among districts (*p* < 0.05).

**Fig 1:**
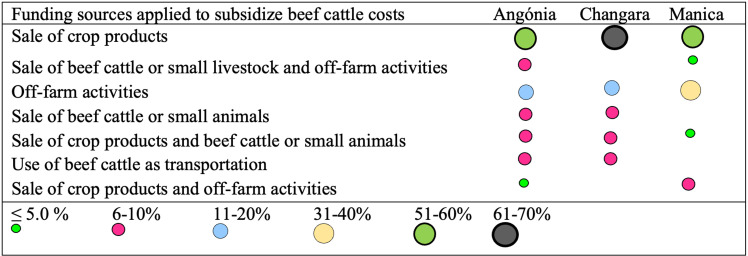
Funding sources to pay cattle production costs (health interventions, feedstuffs, and cattle herder wages) in studied districts of central Mozambique. The number of respondents was 33, 35, and 25 for Angónia, Changara, and Manica, respectively. The sale of crop products versus other sources was not associated with districts (χ^2^ = 1.74, *p* = 0.418).

Crop production aids in supporting beef cattle production. As shown in Fig 1, more than 50% of beef cattle production costs, such as feeding, health, and herding costs are funded by the sale of crop products.

The number of years of experience of cattle farmers was different among districts (*p* < 0.05), with Angónia presenting the lowest median (15 years) (Table 3). Cattle herd size was significantly associated with districts, particularly for herd sizes of less than 11 heads and greater than 20 heads (Table 3). In addition to cattle, farmers rear other livestock species (Table 3).

**Table 3:** Farmers’ years of experience in cattle production, proportional cattle herd size (%), and number (n) of other livestock species reared in beef cattle production systems in the studied districts of central Mozambique.

	Angónia (n = 33)	Changara (n = 37)	Manica (n = 31)	χ^2^	*p*-value
Experience in cattle production (years)					
Mean (SD)	16 (7.72)	21.23 (11.51)	25.1 (12.20)		
Median (Q1-Q3)	15^a^ (9–23.5)	20^b^ (15–25)	25^b^ (15.5–30)		0.011
Min.-Max.	5-30	5-52	5-60		
Cattle, %					
<11 heads	65.67^a^	35.14^b^	77.42^a^	18.74	0.001
11–20 heads	27.27^a^	27.73^a^	16.13^a^		
>20 heads	6.06^a^	35.14^b^	6.45^a^		
^h^ Goats, n					
Mean (SD)	7.2^a^ (5.08)	24.08^b^ (26.61)	8.26^a^ (4.93)		<0.001
^j^ Sheep, n					
Mean (SD)	0.00	12 (9.17)	0.00		
^h^ Pigs, n					
Mean (SD)	4 (2.90)	4.71 (4.71)	6.17 (3.54)		0.264
^h^ Chickens, n					
Mean (SD)	16.04^a^ (13.57)	8.46^b^ (7.19)	18.63^a^ (15.8)		0.010

SD: standard deviation; Q1-Q3: quartiles 1 and 3.

^J^ Sheep were only reared in Changara (8.11% of farmers).

^h^ Calculation based on the total numbers of goats: 25 (Angónia), 21 (Changara), and 23 (Manica); pigs: 11 (Angónia), 6 (Changara), and 7 (Manica); and chickens: 26 (Angónia), 24 (Changara), and 27 (Manica).

^abc^ Values without a common superscript letter in the same row differ among districts (*p* < 0.05).

### 3.2 Cattle acquisition mode at the start of production and historical and current motivations for cattle production

**Fig 2:**
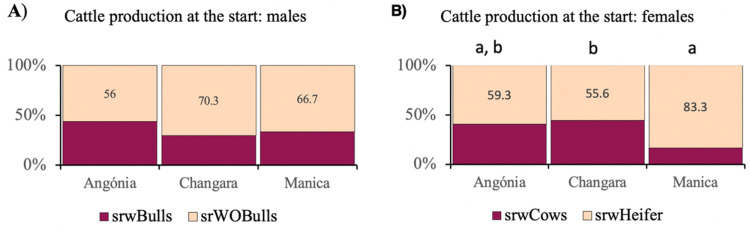
Farmers who started cattle production by purchasing at least one male (**Fig A**, **χ**^**2**^** = 1.63, *p* = 0.503) and females (cows or heifers) (Fig B**, **χ**^**2**^** = 6.27, *p *=** **0.043) in the studied districts of central Mozambique.** srwBulls: started raising beef cattle with their own bulls; srWOBulls: started raising beef cattle without their own bulls; srwCows: started raising beef cattle with cows; srwHeifer: started raising beef cattle with heifers.

Over 83% of farmers started cattle production by purchasing animals (Table 4). Between 56 and 70% of the farmers did not purchase bulls at the start of production (Fig 2A). Those who purchased bulls when starting production intended to use them for draught animal power and not specifically for reproduction. Relative to females, most farmers purchased heifers; however, the proportion between cows and heifers was significantly different among districts (*p* = 0.043) (Fig 2B).

**Table 4:** Mode of acquisition and number of cattle acquired at the start of cattle production in the studied districts of central Mozambique.

	Angónia (n = 33)	Changara (n = 37)	Manica (n = 31)	χ^2^	*p*-value
Mode of cattle acquisition (%)					
Purchase	93.94	94.59	83.87	2.88	0.282^***^
Inheritance	6.06	5.41	16.13		
Initial cattle herd (n)					
Mean (SD)	2.34 (2.51)	3 (2.77)	2 (2.20)		
Median (Q1-Q3)	2 (1-2)	2 (1-4)	2 (1-2)		0.249
Min.-Max.	1-15	1-14	1-12		

SD: standard deviation; Q1-Q3: quartiles 1 and 3.

*** Results of Fisher’s exact test.

**Fig 3:**
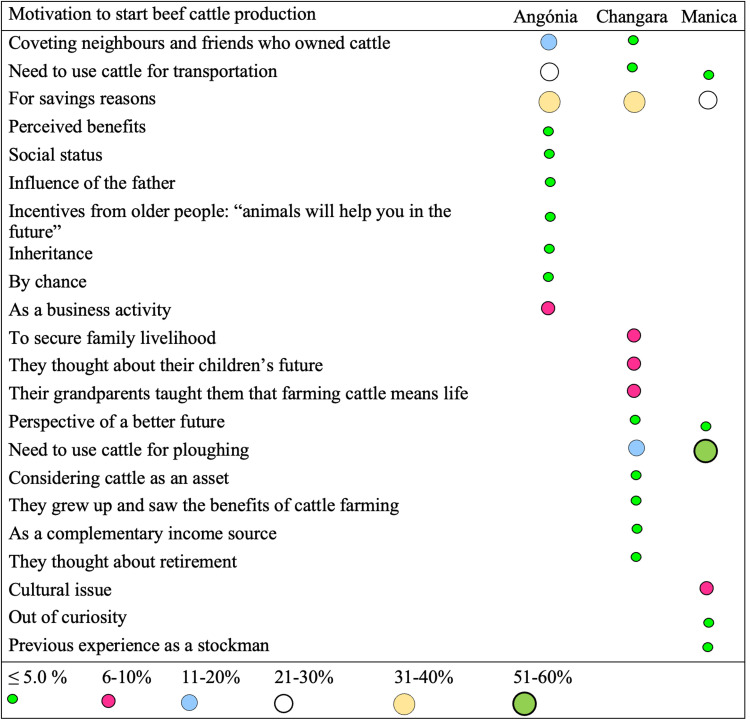
Motivations that influenced the farmers to start beef cattle production in the studied districts of central Mozambique. Savings motivation was independent of districts (χ^**2**^ = 1.952, *p* = 0.377).

The farmers’ motivations to start beef cattle production are shown in Fig 3. In particular, 31–40% of the farmers in Angónia and Changara mentioned savings as their primary motivation, as they could sell the animals during economic hardship, such as illness in the family or lack of food for their household. On the other hand, most farmers in Manica (51–60%) started to produce cattle to use them as draught animals to increase crop production. In Angónia, 21–30% of the farmers mentioned using cattle for transportation as their primary motivation. In all districts, business motivation was only mentioned in Angónia (6–10%).

When savings motivation was analysed, no significant association with districts was detected (S1 Table). Moreover, among the evaluated farmers’ characteristics (age, experience, initial and current cattle herd, and education level), only education level was significantly associated with savings motivation (*p* = 0.028), with a higher proportion of less educated farmers (fifth grade) motivated by savings compared with those with other motivations (seventh grade) (S1 Table).

Over the years, farmers’ motivations did not change (Table 5). However, the mean ranks of the primary motivations differed among districts. The priorities mentioned by the farmers were very similar between Angónia and Manica, with using cattle for draught animal power, savings, and manure ranked 1^st^, 2^nd^, and 3^rd^, respectively, and only differed in milk and meat ranks. Relative to draught animal power, it should be mentioned that in Angónia, all interviewed farmers said they used mainly males for transporting crop products or other products, whereas, in Changara and Manica, both males and females were also used to plough the land.

**Table 5:** Motivations in order of priority of cattle production benefits over the years perceived by the farmers in the studied districts of Central Mozambique.

Motivations	Angónia (n = 33)			Changara (n = 37)			Manica (n = 31)			* *p*-value
n	^A^ Mean (SD)	^1^Rank	n	^A^ Mean (SD)	^1^Rank	n	^A^ Mean (SD)	^1^Rank	
^3^ Draught animal power	28	1.6^a^ (0.74)	1	33	1.67^a^ (0.54)	2	30	1.13^b^ (0.35)	1	<0.001
Savings	33	1.94^ab^ (0.89)	2	37	1.59^b^ (0.93)	1	31	2.14^a^ (0.63)	2	0.011
Manure	32	2.22^a^ (0.71)	3	24	3.46^b^ (0.78)	4	26	2.84^c^ (0.45)	3	<0.001
Milk	5	4.6^a^ (0.89)	5	34	3.0^b^ (0.60)	3	7	3.57^ab^ (0.79)	4	0.002
Meat	11	3.64^a^ (0.92)	4	9	4.44^b^ (1.01)	5	16	3.93^a^ (0.47)	5	0.012

SD: standard deviation

^A^ Mean score on a 1–5 scale.

^1^ The lowest and the highest ranks indicate the most and the least important motivations perceived by the farmers, respectively.

* Results of the Kruskal-Wallis test.

^3^ In Angónia, animal traction is used only for transportation, whereas in Changara and Manica, it is used both for transportation and ploughing the land).

^abc^ Values without a common superscript letter in the same row differ among districts (*p* < 0.05).

In Changara, savings (rank 1) were the primary motivation for cattle production. Another relevant motivation in this district was milk production (rank 3), as most of the interviewed farmers (92%) milked their cows, contrary to Angónia and Manica, where only 15 and 22.58% of the farmers milked their cows, respectively. Since these were beef cows and relied on native communal pastures they were milked mainly between December and April or May, depending on rainfall conditions.

### 3.3 Household participation in cattle grazing activities

Cattle production in the evaluated districts is characterized by the significant participation of the family members (Table 6). The obtained mean ranks show that in Angónia and Changara, hired herders were most frequently responsible for overseeing grazing cattle (rank 1), while in Manica, the farmer’s children ranked first in this activity. Although their mean rank was not statistically different among districts, fathers or owners ranked higher (rank 2) than their children in Angónia, unlike Changara and Manica (rank 3).

**Table 6:** Order of participation of each household member in cattle production activities (taking the animals to the grazing areas daily, see Fig 5) in the studied districts of central Mozambique.

Household member	Angónia (n = 33)			Changara (n = 37)			Manica (n = 31)			* *p*-value
n	^A^ Mean (SD)	1 Rank	n	^A^ Mean (SD)	1 Rank	n	^A^ Mean (SD)	^1^ Rank	
^d^ Herder	30	1^a^ (0.00)	1	30	1^ab^ (0.00)	1	13	1.46^c^ (1.13)	2	0.009
Children	10	2.33^a^ (0.82)	3	35	2.23^ab^ (0.88)	2	28	1.36^c^ (0.56)	1	<0.001
Father or owner	19	2.11 (0.42)	2	24	2.46 (0.51)	3	23	2.35 (0.49)	3	0.24
^k^ Mothers or owner	7	3.57 (0.53)	4	20	3.55 (0.76)	4	19	3.16 (0.60)	4	0.17

SD: standard deviation.

^d^ Cattle herder (worker) age was 15 ± 4 years.

^A^ Mean score from 1 to 4.

^k^ Undertake those activities only when other household members are not available.

^1^ The lowest and the highest ranks indicate the household member who takes the animals to grazing areas the most and the least frequently, respectively.

* Results of the Kruskal-Wallis test.

^abc^ Values without a common superscript letter in the same row differ among districts (*p* < 0.05).

### 3.4 Perception of beef cattle farmers on current pasture condition and availability of water sources for cattle

**Fig 4:**
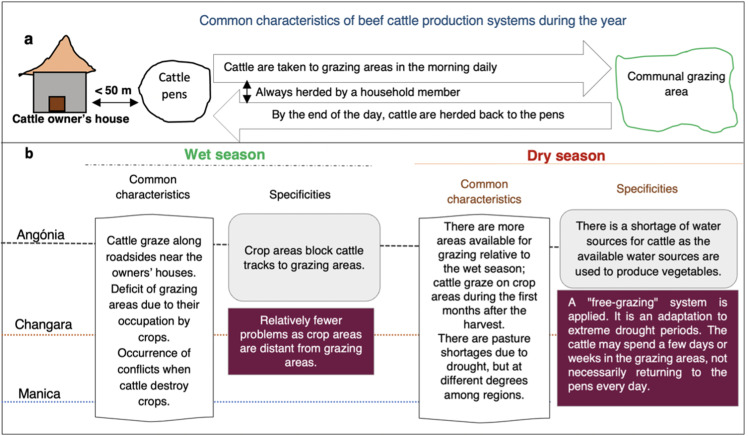
Description of cattle grazing system in the studied districts of central Mozambique. a: indicates the common characteristics throughout the year, and b: indicates the similarities and specificities of each district during different seasons.

**Fig 5:**
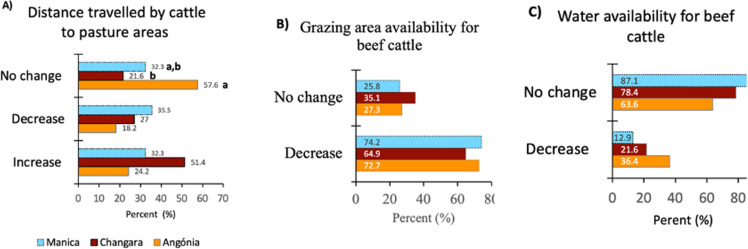
Farmers’ perception of the distance travelled by animals to grazing areas (**χ**^**2**^** = 11.91, *p* = 0.018), availability of grazing areas (χ**^**2**^** = 0.84, *p* = 0.66), and water sources for cattle (χ**^**2**^** = 5, *p* = 0.087) over years of experience in beef cattle production in the studied districts of central Mozambique.**

In all districts grazing areas are predominantly communal and are influenced by season (Fig 4). Farmers’ perceptions of the distance travelled by cattle from the pens to the grazing areas along their years of experience were significantly associated with the evaluated districts (*p* = 0.018; Fig 5A). In Angónia, farmers did not observe any changes in the distance travelled by animals compared with Changara, whereas Manica showed intermediate values. Although farmers’ perception of the increase or decrease in the distance travelled by the cattle was similar among districts, in absolute values, longer distances were perceived in Changara (51.4%) than in Angónia and Manica.

**Fig 6:**
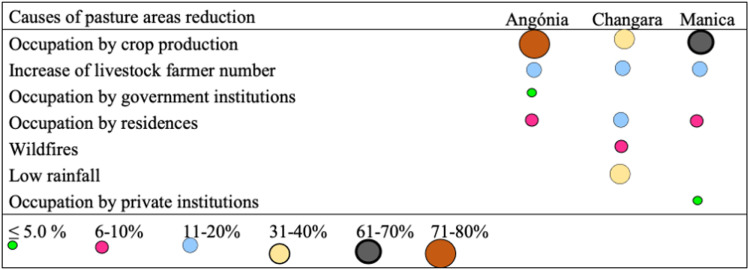
Farmers’ perception of the different causes of grazing area decrease in the studied districts of central Mozambique. The occupation for crop production versus other causes was significantly associated with the districts (χ^2^ = 11.285, *p *= 0.004).

Farmers’ perceptions of the availability of grazing areas were not different among districts (Fig 5B). However, more than 64% of farmers of all districts perceived occupation for crop production as the leading cause of the decrease in grazing areas (Fig 6), and in Changara, low rainfall was considered as important as crop production. Occupation for crop production was perceived as more important than other causes of grazing area decrease by Angónia and Manica farmers than by Changara farmers (*p* = 0.004).

Farmers’ perceptions as to the decrease or absence of changes in the availability of natural drinking water sources for cattle were not different among districts (Fig 5C). However, in absolute values, 36.4% of the farmers in Angónia and only 13% in Changara perceived a decrease in water availability.

## 4 Discussion

In the studied districts of Mozambique, farmers started to produce cattle by purchasing the first animals and in similar numbers. Unlike our results, the inheritance was the primary acquisition mode in Benin, followed by purchase [25]. Our findings may be attributed to the fact that cattle production in those districts has not yet reached sufficient capacity to be transferred to the next generations of a family. Moreover, they suggest that it is still an emerging activity compared to other world regions, where inheritance is the primary acquisition mode.

Farmers’ motivations to engage in different agriculture production activities were analysed by several authors [26–27]. Analysing farmers’ motivation to start producing beef cattle allows an understanding of what drives beef cattle production in the evaluated districts. Among the 22 reasons mentioned by the different farmers, savings was the primary motivation for Angónia and Changara farmers and the second motivation for Manica farmers and may explain the complexity of these systems, considering that the key function of beef cattle production is to supply the continuous demand for beef, both in terms of quantity and quality. When cattle are produced for savings, animals are usually sold when farmers face financial hardship [11]; therefore, such systems do not fulfil that function. Manica farmers started to raise cattle mainly for their contribution to crop production activities (ploughing), indicating a close association of crops with cattle production.

Herd size and years of experience were not statistically different between farmers who started to produce cattle for savings reasons and those with other motivations, indicating that adopting specific management practices was independent of motivation. Moreover, after 5–60 years of experience, despite the policies applied to develop cattle production as a continuous flow income-generating activity, savings and the use of cattle for draught animal power continue to be the farmers’ primary motivations, as previously described in southern Mozambique [11]. The same motivations for producing cattle, notably savings, in Senegal and Malawi [28,29]. However, those authors did not assess the motivations for starting beef cattle production.

The study of labour participation in regions where agriculture is practiced on a small scale is necessary to understand how it can be employed in farming activities [30,31] or even in technological interventions [31]. In the evaluated districts, all household members, not only the hired herder, participate in cattle production activities, as the animals need to be taken to the grazing areas daily. The participation rank of household members in cattle grazing activities differed among districts. For instance, in Angónia and Changara, hired herders ranked first, whereas, in Manica, farmers’ children participated the most. That suggests that it is more challenging to hire herders in Manica due to reduced labour availability, as reported in the South West of England [31]. The greater participation of farmers in herding activities compared with their children in Angónia may be attributed to the small household size in this district. The greater participation of fathers than mothers in herding activities is consistent with the results of other authors [32]. Mothers usually engage in other activities and herd cattle to grazing areas only when other household members are unavailable.

Our results highlight the situation of grazing areas in Mozambique, most of which are communal [33]. Most interviewed farmers in all districts perceived a decrease in grazing areas over the years. Other researchers [29,34] have also observed a decrease in grazing areas in Malawi and Bhutan, respectively. Although that finding may be attributed to multiple causes, the most important is the expansion of crop production area, which may threaten cattle production systems as crops are the primary source of income for cattle farmers in the studied districts.

Relative to the daily distance to grazing areas, Angónia farmers perceived fewer changes over the years than those in Changara and Manica. In addition, despite not being statistically different among districts, a higher percentage of Changara farmers (51.4%) considered the distance increased. An increase in the distance to grazing areas over the years was perceived by 70% of farmers in Malawi [29]. The lower percentage obtained in the present study relative to Malawian farmers suggests that this issue is less pronounced in the evaluated districts and that interventions may have promising outcomes.

The finding that farmers perceived a decrease in the distance travelled to grazing areas and no changes in grazing area availability may be explained by their migration to other areas. Migration could be an alternative cattle production model; however, it may be complex to implement, as cattle pens are commonly located close to the farmers’ houses (Fig 4), and crops are their main income source. Moreover, migrating to other areas implies a series of structural, social, and economic changes, which cattle farmers motivated by savings may not be willing to make. This perception may change if State policies providing subsidies to farmers are established. Those results suggest that production improvement interventions should focus on preserving grazing areas, as they are a vital resource for cattle production, and be directed to cattle farmers who consider this activity a business opportunity despite having other motivations.

In general, the results of this study emphasize the challenges of developing policies to improve the beef cattle production systems because farmers do not perceive cattle production as an economic activity *per se*. Since farmers perceive draught animal power and savings as the main benefits of cattle production, policy development should adopt a multidisciplinary approach. In particular, cattle farmers need to be shown that, by reorganizing their production systems, they can obtain higher prices for the cattle sold during financial hardship.

## 5 Conclusions

The past and current motivations for cattle production in the studied locations are associated with the benefit achieved by cattle farmers at times of greater financial need, indicating that, for market-orientated production, it is necessary to redesign strategies to promote beef production based on an in-depth understanding of the “farmer factor.” The farmers’ perceptions about grazing areas indicate the need for strategies to improve the management of both grazing and crop areas.

## Supporting information

S1 Table Farmers’ characteristics according to the motivation to start cattle production in the studied districts of central Mozambique. (DOCX)

S1 File Human Participants Research Checklist. (DOCX)
