# Supplementary material for: An understanding of the motivations that influence the beef cattle production systems adopted by farmers in central Mozambique
Source: PLoS One. 2025 Jun 17;20(6):e0325929. doi: 10.1371/journal.pone.0325929 (PMC12173388; doi:10.1371/journal.pone.0325929)
Supplement: S1 Table — (DOCX) [file pone.0325929.s001.docx]

**S1 Table. Farmers’ characteristics according to the motivation to start cattle production in the studied districts of central Mozambique.**

|  |  | Savings motivation (n=31) | |  | Non-savings motivation (n=71) | | ^5^*p*-value |
| --- | --- | --- | --- | --- | --- | --- | --- |
|  | Unit | Mean (SD) | Median (Q1-Q3) |  | Mean (SD) | Median (Q1-Q3) |  |
| Experience in cattle production | Year | 21.17 (12.54) | 22 (11-27) |  | 20.61 (10.36) | 19 (14.75-25) | 0.921 |
| Age |  | 51.94 (12.54) | 51 (42-60) |  | 51.5 (12.46) | 50 (42-59.8) | 0.819 |
| Education | Grades | 5.19 (2.71) | 5 (3-7) |  | 6.47 (2.73) | 7 (4-8) | 0.028** |
| Cattle | herd | 13 (11.31) | 11 (6-17) |  | 13.27 (14.53) | 8 (5.75-15) | 0.526 |
| Initial cattle |  | 2.9 (3.26) | 2 (1-3) |  | 2.39 (2.1) | 2 (1-2.25) | 0.889 |

SD: standard deviation; Q1-Q3: quartiles 1 and 3.

^5^*p*-value of the Mann-Whitney U test. **p <0.05.
